# Association of mercury exposure with the serum high-sensitivity C-reactive protein level in Korean adults

**DOI:** 10.3389/fpubh.2023.1062741

**Published:** 2023-03-28

**Authors:** Kisok Kim, Hyejin Park

**Affiliations:** ^1^College of Pharmacy, Keimyung University, Daegu, Republic of Korea; ^2^Department of Health Sciences, Dongduk Women’s University, Seoul, Republic of Korea

**Keywords:** cardiovascular disease, high-sensitivity C-reactive protein, inflammation, KNHANES, mercury

## Abstract

Although there is evidence that mercury (Hg) exposure may be a potential risk factor for cardiovascular disease (CVD), few nationwide epidemiological researches have analyzed the association between blood Hg concentration and serum high-sensitivity C-reactive protein (hs-CRP) level as a biomarker of CVD. The present population-based national study was performed with data from the 2016–2017 National Health and Nutrition Examination Survey. In the total sample of 3,773 adults aged ≥20 years, the serum hs-CRP concentrations were 1.03 mg/L among participants in the lowest quartile of blood Hg level and 1.18 mg/L among those in highest quartile. The trend for the prevalence of a risky (>1.0 mg/L) hs-CRP level (moderate risk and high risk) was significantly related to an increased quartile blood Hg concentration. After adjustment for confounders, participants with the highest quartiles of blood Hg had increased odds of a risky (>1.0 mg/L) hs-CRP level (adjusted odds ratio = 1.34; 95% confidence interval, 1.02–1.77) compared with those with the lowest quartile of blood Hg. These findings demonstrate that a high blood Hg level increases the concentration of serum hs-CRP, a sensitive marker of chronic low-grade inflammation, and imply that the increased body burden associated with high blood Hg is a potential risk factor in the development of many inflammatory diseases, including CVD.

## Introduction

Cardiovascular disease (CVD) is the leading cause of disability and death worldwide, and the numbers of CVD cases and deaths continue to rise (1). In Korea, although the prevalence has been declining in recent years, CVD is still the second main cause of mortality (2). In addition to traditional risk factors for CVD, such as hypertension, diabetes, obesity, and high salt consumption, there are growing concerns about environmental toxicants, such as toxic metals and persistent organic pollutants, as a potential risk factor for CVD (3, 4). In particular, potentially toxic metals are widely distributed in the ambient air, drinking water, and foods; thus, the adverse health effects of toxic metal exposure are of great concern to many population groups globally (5).

Among metals, mercury (Hg) is a toxic metal, with known harmful effects on human health following exposure (6). The general population may be exposed to Hg through environmental or occupational exposure sources; additionally, consumption of fish/seafood is the most common route of exposure to methylmercury (MeHg), a highly toxic form of Hg (7). Hg exposure is recognized as an important public health issue, as Hg is reported primarily as a metal that causes neurodevelopmental toxicity in neonates and children (8, 9). However, there is a growing body of epidemiological and experimental evidence indicating that Hg exposure may increase the risk of adverse cardiovascular outcomes (10–12).

Although the mechanisms supporting the association between Hg exposure and CVD prevalence have not been elucidated in detail, there are some reports that Hg exposure impairs the activity of selenoproteins and promotes lipid peroxidation, as well as chronic inflammation (13). As chronic inflammation is the critical mechanism in the pathogenesis of CVD (14) and serum high-sensitivity C-reactive protein (hs-CRP) is an indicator of inflammation (15–18), hs-CRP is considered to be a biomarker of CVD risk (19).

The identification of environmental risk factors influencing the incidence of CVD in the general population is important for the reduction of disability and premature death from CVD. However, few studies have examined the association between blood concentration of Hg and the hs-CRP level, as a sensitive marker of CVD risk, in the general Korean population. Therefore, the present study was conducted to evaluate this association in Korean adults using data from the Korea National Health and Nutrition Examination Survey (KNHANES).

## Materials and methods

### Study population

**Figure 1:**
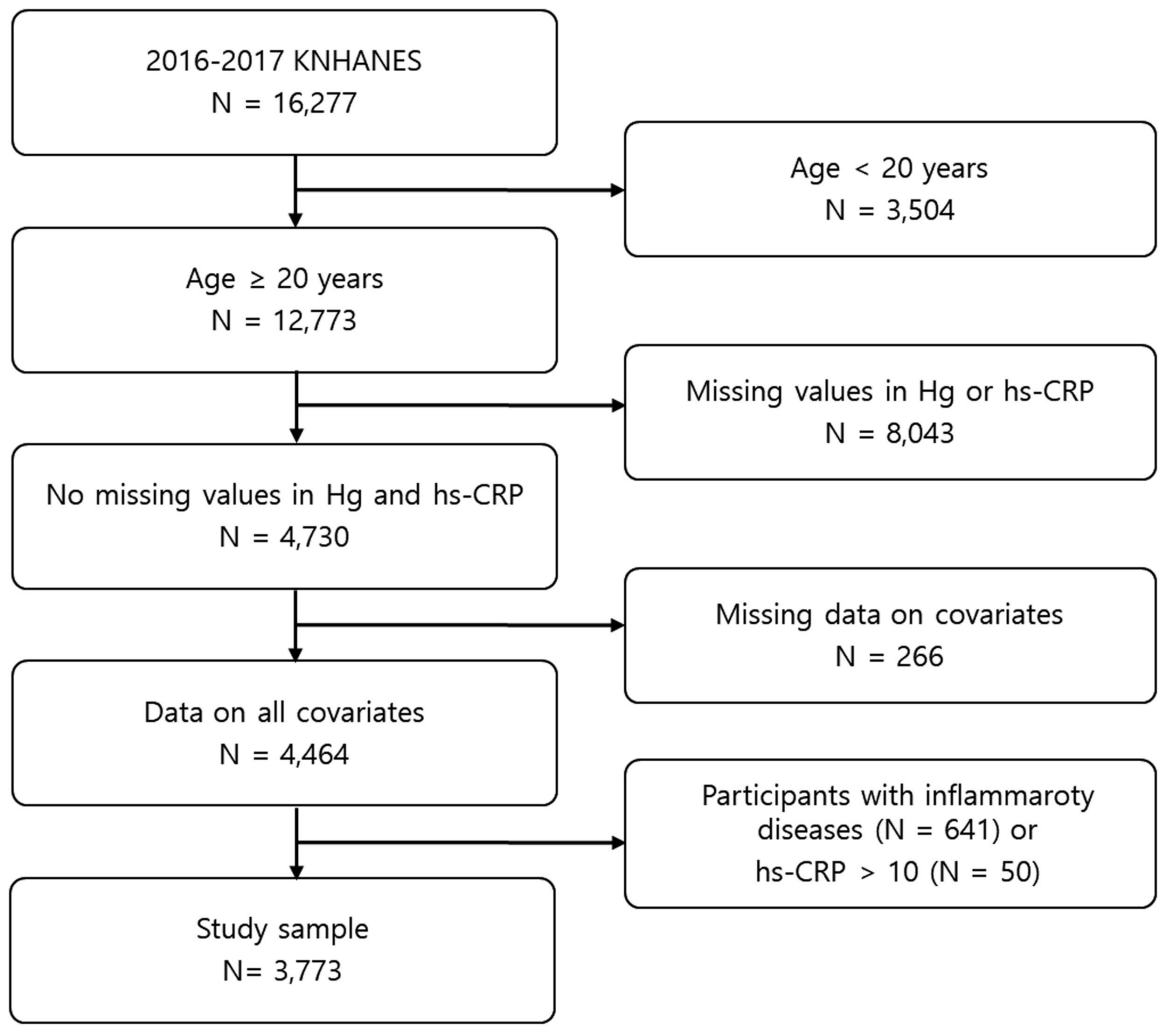
Flow chart of study sample selection.

This study was carried out based on the data obtained from the 2016–2017 KNHANES, provided by the Korea Disease Control and Prevention Agency. The KNHANES is performed by applying a stratified multi-stage cluster sampling design, with proportional allocation based on the national census register, to acquire a nationally representative sample of the general Korean population. Because inflammatory conditions can affect the hs-CRP level, participants with thyroid disease, which may induce inflammation, and participants with inflammatory diseases such as asthma and arthritis were excluded (*n* = 641). In addition, because hs-CRP levels higher than 10 mg/L are likely indicative of infection, participants with a hs-CRP > 10 mg/L were excluded (*n* = 50). Ultimately, a sample of 3,773 adults aged ≥20 years was analyzed in this study (Figure 1). The protocol of this study was approved by the Korean Ministry of Health and Welfare, and the research was conducted in accordance with the principles of the Declaration of Helsinki. All study participants provided informed consent.

### Data collection

In the KNHANES, a questionnaire was used to obtain data on the sociodemographic characteristics of the participants, including sex, age, education level, income, cigarette smoking status, and alcohol drinking status. Education level was classified as less than high school, high school, and more than high school. Income was obtained by dividing the total household income by the square root of the household size. Data on alcohol drinking were acquired by asking questions related to alcohol drinking habits (average alcohol drinking frequency) in the month before the interview. Cigarette smokers and alcohol drinkers were defined as respondents who reported current cigarette smoking and alcohol drinking at least once a month, respectively. Based on the measured height and weight of the participant, the body mass index (BMI) was calculated by using weight in kilograms (kg) divided by the square of height in meters (m^2^). Then, participants were classified as underweight (BMI < 18.5 kg/m^2^), normal weight (18.5 kg/m^2^ ≤ BMI < 23.0 kg/m^2^), overweight (23.0 kg/m^2^ ≤ BMI < 25.0 kg/m^2^), and obese (BMI ≥ 25.0 kg/m^2^) according to the World Health Organization’s criteria for Asian individuals. Total Hg in blood was measured using a cold-vapor atomic absorption spectrometric method and a dedicated Hg analyzer (M-6000A; CETAC Technologies, Omaha, NE, United States). The details of the Hg analysis procedures have been published elsewhere (20). For the analysis of serum hs-CRP, a COBAS analyzer (Roche Diagnostics, Basel, Switzerland) and an immunoturbidimetric assay (Roche Cardiac C-Reactive Protein High Sensitive Reagent Kit) were used. The analytical measurement range of hs-CRP assay was 0.1–20 mg/L. In this study, moderate-to-high risk hs-CRP levels (>1 mg/L), as specified by the American Heart Association (21), were defined as risky hs-CRP levels.

### Statistical analysis

To account for the sample population studied, we calculated frequencies and percentages for sociodemographic variables. In addition, geometric means with 95% confidence intervals (CIs) for blood Hg concentration were calculated by taking the anti-log of the arithmetic mean of log-transformed values. Based on a normal probability distribution, we improved the approximation of a normal distribution using the geometric mean. The Mantel–Haenszel chi-squared test was used to test the significance of differences for categorical variables. Multivariate logistic regression analysis was performed to estimate odds ratios (ORs) with 95% CIs for risky hs-CRP status [vs. low risk (hs-CRP < 1.0 mg/L)] among quartiles of Hg concentration, after adjusting for possible confounding variables such as age, BMI, education level, income, cigarette smoking, and alcohol drinking. The Cochran-Armitage test was used to determine the presence of a significant linear trend in the prevalence of a risky hs-CRP level across the Hg quartiles. The statistical analyses were conducted, taking into account the survey design, and appropriate statistical procedures in SAS (e.g., “surveylogistic”) were applied with weighted data. All statistical analyses were performed using SAS software version 9.4 (SAS Institute Inc., Cary, NC, United States).

## Results

The study sample consist of 3,773 adults aged ≥20 years; their sociodemographic characteristics are presented in Table 1. The average age of the study participants was 48.3 years, and the mean BMI was 23.9 kg/m^2^.

**Table 1:** Demographic characteristics of the study participants.

Characteristics	*N*	%
Sex		
Male	1802	47.8
Female	1971	52.2
Age (years)		
20–39	1,252	33.2
40–59	1,563	41.4
≥60	958	25.4
BMI		
<18.5	153	4.1
18.5–22.9	1,457	38.6
23.0–25.0	848	22.5
>25.0	1,315	34.8
Education		
<High school	898	23.8
High school	1,280	33.9
> High school	1,595	42.3
Income (US$/month)		
<690	897	23.8
690–1,419	959	25.4
1,420–2,320	912	24.2
>2,320	1,005	26.6
Cigarette smoking		
Yes	791	21.0
No	2,982	79.0
Alcohol drinking		
Yes	2,573	68.2
No	1,200	31.8

BMI, body mass index.

The geometric mean blood Hg level is presented according to the participants’ demographic characteristics in Table 2. Of the 3,773 participants, the geometric mean blood Hg level was 3.28 μg/L; the Hg concentration in men was significantly higher than that in women (*p* < 0.001). The geometric mean blood Hg level increased with BMI, education level, and income (*p* < 0.05). In addition, age, cigarette smoking, and alcohol drinking were significantly associated with blood Hg (*p* < 0.001).

**Table 2:** Blood Hg concentrations by demographic characteristics in Korean adults aged ≥20 years.

Characteristics	*N*	Geometric mean Hg [μg/L (95% CI)]	*P*-value^a^
Total	3,773	3.28 (3.22–3.35)	
Sex			<0.001
Male	1802	3.97 (3.85–4.09)	
Female	1971	2.76 (2.69–2.83)	
Age (years)			<0.001
20–39	1,252	2.93 (2.83–3.02)	
40–59	1,563	3.60 (3.49–3.72)	
≥60	958	3.28 (3.14–3.43)	
BMI			<0.001
<18.5	153	2.66 (2.42–2.92)	
18.5–22.9	1,457	2.93 (2.84–3.02)	
23.0–27.4	848	3.59 (3.45–3.75)	
≥27.5	1,315	3.60 (3.48–3.73)	
Education			0.040
<High school	898	3.16 (3.03–3.30)	
High school	1,280	3.18 (3.07–3.29)	
>High school	1,595	3.44 (3.34–3.55)	
Income (US$/month)			<0.001
<690	897	2.97 (2.85–3.09)	
690–1,419	959	3.18 (3.06–3.31)	
1,420–2,320	912	3.31 (3.18–3.45)	
>2,320	1,005	3.68 (3.53–3.83)	
Cigarette smoking			<0.001
Yes	791	3.89 (3.73–4.06)	
No	2,982	3.14 (3.07–3.21)	
Alcohol drinking			<0.001
Yes	2,573	3.53 (3.44–3.62)	
No	1,200	2.81 (2.72–2.91)	

a *P*-values from the Mantel–Haenszel chi-squared test for the difference in geometric mean mercury (Hg) concentrations between groups.

**Figure 2:**
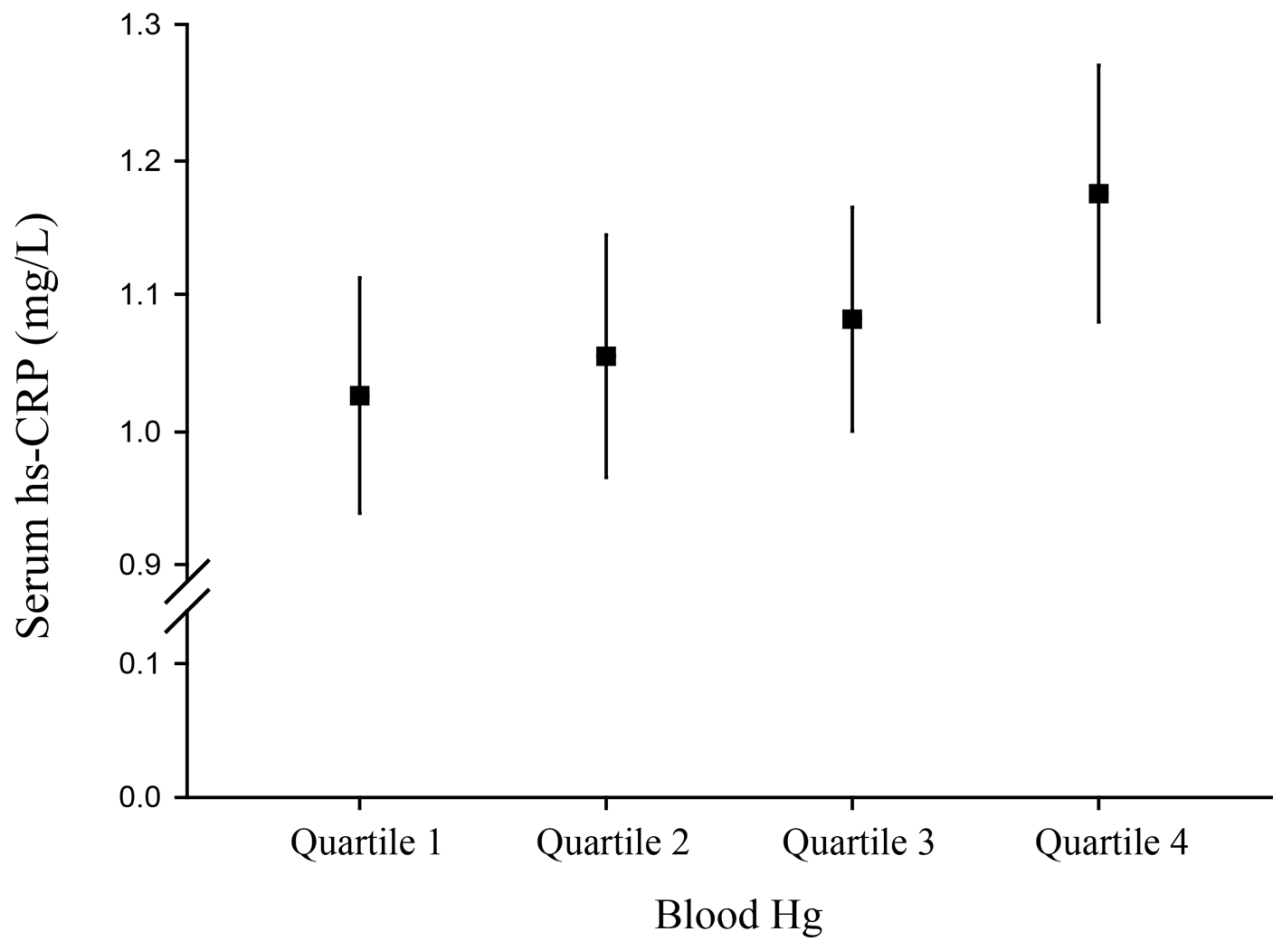
Serum high-sensitivity C-reactive protein (hs-CRP) level (95% confidence interval) by quartile of blood Hg.

The serum hs-CRP level was significantly associated with an increased quartile of blood Hg concentration (*p* for trend = 0.019; Figure 2). The serum hs-CRP concentration was 1.18 (95% CI, 1.08–1.27) mg/L among participants having the highest quartile Hg compared to 1.03 (95% CI, 0.94–1.11) mg/L among those having the lowest quartile Hg.

Table 3 shows the prevalences and ORs for the associations of having a risky hs-CRP level (moderate-to-high level) with respect to quartiles of Hg concentration. The prevalence of a having a hs-CRP level > 1.0 mg/L was higher among participants in the higher quartile Hg concentration: 26.1% among participants with the lowest quartile Hg (<2.14 μg/L), and 32.0% among those with the highest quartile Hg (> 4.89 μg/L) (*p* = 0.003). The crude and adjusted ORs (Models 1 and 2) for a risky hs-CRP level was positively correlated with an increasing quartile Hg level (*p* < 0.01). The crude OR of a risky hs-CRP level in the highest quartile Hg group (blood Hg level > 4.89 μg/L) relative to the reference group (blood Hg level < 2.14 μg/L) was 1.56 (95% CI, 1.23–1.99), and the fully adjusted OR for a risky hs-CRP level was 1.34 (95% CI, 1.02–1.77) (Model 2).

**Table 3:** Prevalences and adjusted odds ratios (95% confidence intervals) of a risky hs-CRP level by quartile of blood Hg in Korean adults aged ≥20 years.

Risky hs-CRP	Quartile blood Hg level (μg/L)				*P* for trend
	Quartile 1 (<2.14)	Quartile 2 (2.14–3.19)	Quartile 3 (3.20–4.89)	Quartile 4 (>4.89)	
Prevalence, %	26.11	27.7	29.52	32.0	0.003
OR (95% CI)					
Crude	1.00 (reference)	1.14 (0.89–1.45)	1.25 (0.97–1.62)	1.56 (1.23–1.99)	0.004
Model 1	1.00 (reference)	1.13 (0.88–1.44)	1.17 (0.90–1.52)	1.39 (1.07–1.79)	<0.001
Model 2	1.00 (reference)	1.10 (0.85–1.42)	1.14 (0.87–1.50)	1.34 (1.02–1.77)	<0.001

hs-CRP, high-sensitivity C-reactive protein; Model 1, adjusted for age and sex; Model 2, adjusted for age, sex, BMI, education, income, cigarette smoking status, and alcohol drinking status.

## Discussion

Hg is considered to be one of the most widespread toxic environmental pollutants, and exposure to Hg can occur from both natural and artificial sources (22). Humans are exposed to Hg through several sources including environmental pollutants, contaminated seafood, metallic Hg-containing dental amalgam, and ethyl-Hg-containing vaccines (23, 24). Hg has possible toxic effects on the nervous, digestive, and immune systems, as well as renal function (25, 26). However, accumulating evidence suggests that exposure to Hg is contribute to the risk of CVD and related outcomes, including hypertension and ischemic heart disease (IHD) (11, 12, 27). A meta-analysis of 14 studies reported that chronic exposure to Hg was associated with an increased risk of fatal/nonfatal IHD and all-cause mortality (28). However, in a cohort study of adults in the United States (US), blood Hg level was not significantly related to the risk of all-cause or CVD-related mortality (29). Another population-based cohort study conducted in Greenland also reported no association between blood Hg and the risk of developing CVD (30). Therefore, a potential biomarker associated with CVD could be used to better understand the causal relationship between Hg exposure and CVD prevalence.

Low-grade inflammation is a known contributor to the pathogenesis of several chronic diseases, including CVD, and the use of hs-CRP as a sensitive marker for low-grade inflammation has been investigated extensively (31–33). Several cohort studies have reported that an elevated hs-CRP level is independently associated with an increased incidence of major cerebrovascular and cardiovascular outcomes (34, 35). Similarly, many recent studies have shown that the hs-CRP level is positively correlated with CV risk in various clinical settings (36–39).

In this study, an increase in the blood Hg concentration was significantly associated with an increase in the hs-CRP concentration. Acute exposure to Hg from dental amalgam was reported to have no significant effect on the level of C-reactive protein (CRP) (40), but the association between blood Hg and the level of CRP has been reported in some epidemiological studies (41, 42). In a study of premenopausal women (mean age, 27.4 years) in the US, although it was not statistically significant, a positive association was observed between Hg concentration and hs-CRP level with an adjusted beta coefficient of 0.02 (41). In addition, in the NHANES 2005–2006 study conducted with US participants aged 16–49 (mean age, 34 years), the beta coefficient between Hg and CRP levels was positive in a fully adjusted multiple linear regression model in women, but not in men (42). These two previous studies of US participants showed a positive correlation between Hg and CRP levels only in women and did not reach statistical significance. In our study of Korean adults, a high level of Hg exposure significantly increased the hs-CRP concentration and the frequency of having a risky (> 1.0 mg/L) hs-CRP level. Specifically, participants with the highest quartile of blood Hg were more likely than those with the lowest quartile to have an hs-CRP level > 1.0 mg/L (adjusted OR = 1.34; 95% CI, 1.02–1.77); the cutoff value is associated with the CVD risk. The mean age of our study population was 48.3 years; the difference between the OR values found in our study and those reported in the US studies may be due, at least in part, to the difference in the average age of the study subjects. Given that the geometric mean of blood Hg concentration was much higher in Korean participants than in US participants (3.28 μg/L vs. 1.05 μg/L), the blood Hg level may also have affected the statistical significance of the results. Additionally, differences in race and ethnicity between Korean and US participants likely influenced the findings.

Few epidemiological studies have examined the associations between body burden of Hg and hs-CRP level. To the best of our knowledge, this study is the first to elucidate the relationship between Hg exposure and hs-CRP level, as a highly sensitive cardiovascular biomarker, using population-based survey data on Korean adults aged ≥20 years. However, there are several limitations to this study. First, given the complexity of potential factors affecting serum hs-CRP concentrations, additional factors, including oxidative stress and inflammatory comorbidities, which were not fully considered in the model of this study, could have significant effects on serum hs-CRP (43). Therefore, future studies should consider factors such as antioxidant food or supplement consumption; the use of anti-inflammatory drugs; and the prevalence of inflammatory diseases such as cancer, inflammatory bowel disease, chronic kidney disease, and type 2 diabetes. Second, several sociodemographic factors, including alcohol drinking and cigarette smoking, were derived from self-reporting and therefore may have introduced misclassification or recall bias. Third, although we adjusted for potential confounders in the analysis, several confounding factors, such as vitamins and trace elements that are associated with inflammatory markers and may interact with Hg, were not adjusted for in the study. Despite these limitations, our study findings are important in view of a nationwide study that found an association between body burden of Hg and the potential for chronic low-grade inflammation that can lead to CVDs. Due to the limitation of the cross-sectional design of our study, prospective cohort studies are warranted to elucidate a causal relationship between Hg exposure and cardiovascular markers such as hs-CRP.

## Conclusion

In the present study using nationally representative survey data on Korean adults aged ≥20 years, an increase in blood Hg concentration increased the low-grade inflammatory response, as reflected in the hs-CRP level. The results of this study show that Hg exposure may increase chronic inflammation, which in turn may lead to an increased risk of inflammatory diseases such as CVD, type 2 diabetes, and cancer.

## Data availability statement

Publicly available datasets were analyzed in this study. This data can be found at: https://knhanes.kdca.go.kr/knhanes/eng/index.do.

## Ethics statement

The studies involving human participants were reviewed and approved by Korean Ministry of Health and Welfare. The patients/participants provided their written informed consent to participate in this study.

## Author contributions

KK: conceptualization, methodology, investigation, and writing–review and editing. HP: investigation, data curation, and writing–original draft. All authors have read and agreed to the published version of the manuscript.

## Funding

This research was supported by the Keimyung University Research Grant of 2019.

## Conflict of interest

The authors declare that the research was conducted in the absence of any commercial or financial relationships that could be construed as a potential conflict of interest.

## Publisher’s note

All claims expressed in this article are solely those of the authors and do not necessarily represent those of their affiliated organizations, or those of the publisher, the editors and the reviewers. Any product that may be evaluated in this article, or claim that may be made by its manufacturer, is not guaranteed or endorsed by the publisher.
